# Structure Elucidation and Cytotoxic Evaluation of New Polyacetylenes from a Marine Sponge *Petrosia* sp.

**DOI:** 10.3390/ijms150916511

**Published:** 2014-09-18

**Authors:** Yung-Shun Juan, Chien-Chih Lee, Chia-Wei Tsao, Mei-Chin Lu, Mohamed El-Shazly, Huei-Chuan Shih, Yu-Cheng Chen, Yang-Chang Wu, Jui-Hsin Su

**Affiliations:** 1 Department of Urology, Kaohsiung Municipal Hsiao-Kang Hospital, Kaohsiung 812, Taiwan; E-Mail: juanuro@gmail.com; 2 Department of Urology, College of Medicine, Kaohsiung Medical University, Kaohsiung 807, Taiwan; 3 Department of Urology, Kaohsiung Medical University Hospital, Kaohsiung 807, Taiwan; 4 Epigenome Research Center, Department of Laboratory Medicine, China Medical University Hospital, Taichung 404, Taiwan; E-Mail: insect@hotmail.com.tw; 5 School of Chinese Medicine, College of Chinese Medicine, China Medical University, Taichung 404, Taiwan; 6 Graduate Institute of Marine Biotechnology, National Dong Hwa University, Pingtung 944, Taiwan; E-Mails: gaway4297@yahoo.com.tw (C.-W.T.); jinx6609@nmmba.gov.tw (M.-C.L.); j520c@hotmail.com (Y.-C.C.); 7 National Museum of Marine Biology and Aquarium, Pingtung 944, Taiwan; 8 Department of Pharmacognosy and Natural Products Chemistry, Faculty of Pharmacy, Ain-Shams University, Organization of African Unity Street, Abassia, Cairo 11566, Egypt; E-Mail: elshazly444@googlemail.com; 9 Department of Nursing, Meiho University, Pingtung 912, Taiwan; E-Mail: x00002213@meiho.edu.tw; 10 School of Pharmacy, College of Pharmacy, China Medical University, Taichung 404, Taiwan; 11 Chinese Medicine Research and Development Center, China Medical University Hospital, Taichung 404, Taiwan

**Keywords:** sponge, *Petrosia*, polyacetylene

## Abstract

The sponge *Petrosia* sp. yielded five polyacetylenic compounds (**1**–**5**), including two new polyacetylenes, petrosianynes A (**1**) and B (**2**). The structures of these compounds were elucidated by detailed spectroscopic analysis and by comparison with the physical and spectral data of related known analogues. Compounds **1**–**5** exhibited significant cytotoxic activity against a limited panel of cancer cell lines.

## 1. Introduction

**Figure 1:**
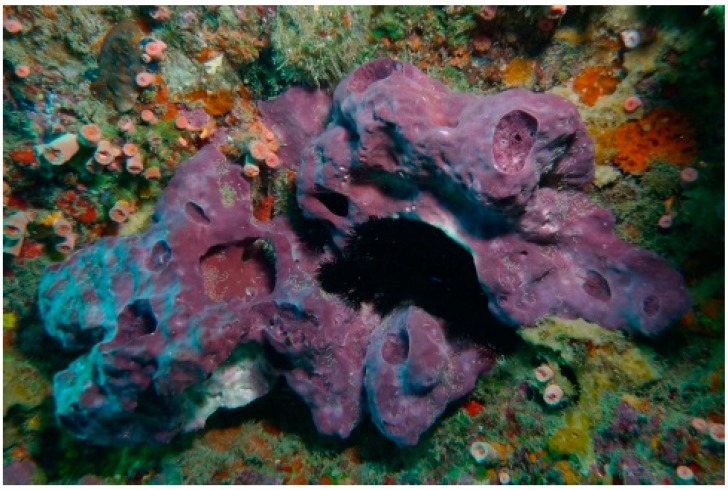
Sponge *Petrosia* sp.

**Figure 2:**
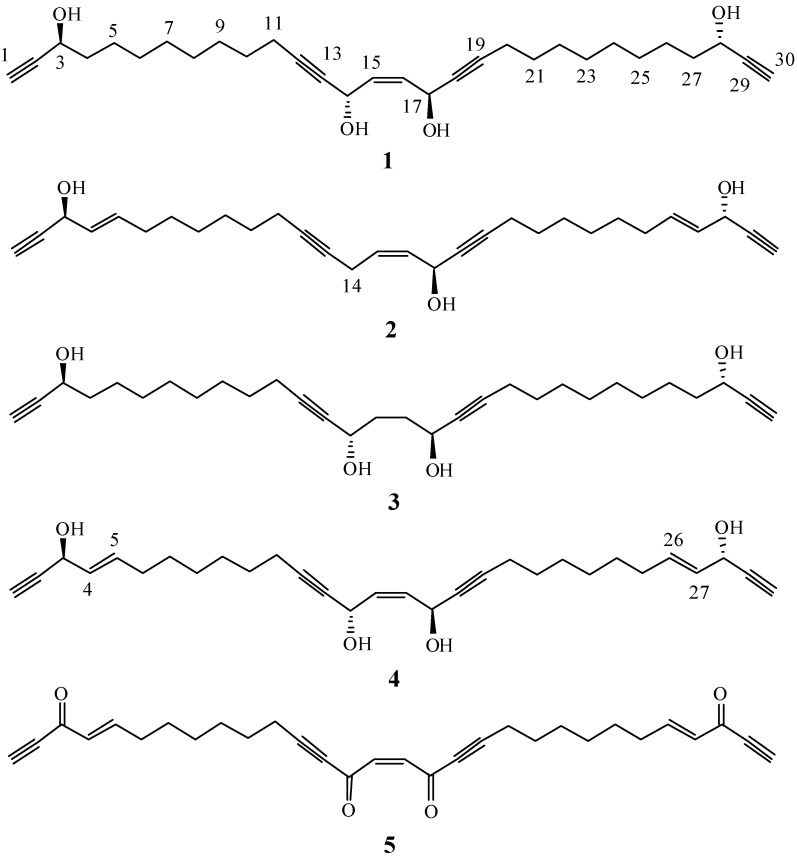
Structures of the isolated metabolites **1**–**5**.

Since the early days of marine natural products chemistry, sponges (Porifera) have occupied a superior place in the focus of researchers aiming to reveal the therapeutic potentials of these benthic organisms [1]. Thousands of secondary metabolites have been identified from these organisms and their contributions to the chemical library continue with fascinating results. Such a sponge-centric theme comes as no surprise, because such organisms have been spotted since antiquity and their unique secondary metabolites suggested a myriad of potential applications [2,3,4]. Among the well-studied Porifera genera is *Petrosia* sp. They have been subjected to intensive scrutiny following the separation of different acyclic polyacetylenes with potent biological activities from sponges belonging to this genus [5,6,7]. Despite the fact that certain acyclic polyacetylenes can be found in terrestrial plants, especially the family Asteraceae, *Petrosia* sp. have been known to be a source of polyacetylenes with unique structural features as it has been demonstrated over the last two decades of research [8]. It is estimated that 33% of all known polyacetylenes have been isolated from *Petrosia* sp. [9]. In addition to the unique structural features of the isolated polyacetylenes, a plethora of interesting biological activities has been reported for this class of secondary metabolites, including antimicrobial [10], antifungal [11,12], reverse transcriptase inhibitory [13] and antitumor activities [9,14,15]. In a search for bioactive metabolites from marine organisms, the sponge *Petrosia* sp. (Figure 1) was selected for a detailed investigation, as its EtOAc crude extract showed significant cytotoxicity in six human tumor cell lines. Bioassay-guided fractionation resulted in the isolation of two new polyacetylenic compounds (**1** and **2**) and three known polyacetylenes (**3**–**5**) (Figure 2). The cytotoxic activity of the metabolites (**1**–**5**) against human T cell lymphoblast-like cell line (CCRF-CEM), human T lymphoblast, acute lymphoblastic leukemia (MOLT-4), human chronic myelogenous leukemia (K-562), human colon adenocarcinoma (DLD-1), human prostate carcinoma (LNCaP) and human hormone-dependent breast cancer (T-47D) cell lines was evaluated.

### 2. Results and Discussion

Following the chromatographic separation of *Petrosia* sp., the EtOAc soluble fraction yielded five polyacetylenic compounds, two of which were new natural products (**1** and **2**). All compounds were obtained as colorless oils. The new compounds were given the trivial names petrosianynes A (**1**) and B (**2**). Three compounds **3**–**5** were found to be identical to the known polyacetylenes 15,16-dihydropetrosianyne (**3**) [16], petrosynol (**4**) [17] and petrosynone (**5**) [10], respectively, through the comparison of their physical and spectroscopic data with those reported in the literature.

**Figure 3:**
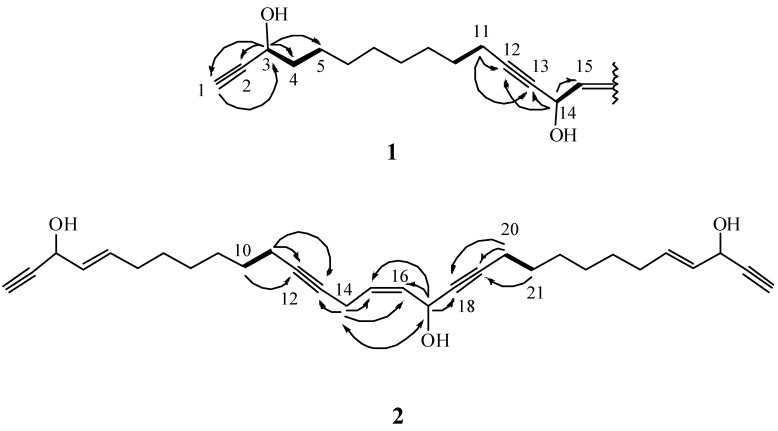
Key ^1^H–^1^H COSY (▬), HMBC (→) and NOE (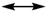) correlations of **1** and **2**.

Compound **1**, which was isolated as a colorless oil, was found to have the molecular formula C_30_H_44_O_4_ deduced by HRESIMS at *m*/*z* 491.3135 [M + Na]^+^. The IR spectrum of **1** showed absorption bands due to a hydroxyl (3425 cm^−1^) and alkyne (3280 and 2225 cm^−1^) moieties. The MS, ^1^H and ^13^C NMR spectra (Table 1) of **1** showed characteristic signal patterns that were reminiscent of the C2-symmetrical structure. The planar structure and all of the ^1^H and ^13^C chemical shifts of **1** were elucidated by 2D NMR spectroscopic analysis, especially the ^1^H–^1^H COSY and HMBC correlations (Figure 3). Thus, **1** was found to possess two terminal acetylenes at C-1/C-2 and C-29/C-30, two disubstituted acetylenes at C-12/C-13 and C-18/C-19, one double bond at C-15/C-16 and four hydroxy groups at C-3, C-14, C-17 and C-28. In addition, the spectroscopic data of **1** (IR, ^1^H and ^13^C NMR) were similar to those of **4**, which was isolated from the same sponge. Comparison of the ^1^H and ^13^C NMR data of **1** with **4** [17] showed that the signals corresponding to a 1,2-disubstituted double bond in **4** were replaced by signals of a single bond in **1**. As the new metabolite **1** was isolated together with **3** [16] and **4** from the same species and possesses a similar molecular skeleton, it was proposed that the three compounds are synthesized through a common biosynthetic pathway and thus have the same absolute configurations at C-3, C-14, C-17 and C-28. On the basis of the above analyses, the structure of **1** was established and the compound was named petrosianyne A. 

**Table 1:** ^1^H and ^13^C NMR data for **1** and **2**.

Position	1		2	
	δ_H_ (*J* in Hz) *^a^*	δ_c_ (mult.) *^b^*	δ_H_ (*J* in Hz) *^a^*	δ_c_ (mult.) *^b^*
1	2.47 d (2.0)	72.8 (CH)	2.57 d (2.0)	74.0 (CH)
2		85.0 (C)		83.3 (C)
3	4.38 ddd (6.5, 6.5, 2.0)	62.3 (CH)	4.84 d (6.0)	62.8 (CH)
4	1.72 m	37.6 (CH_2_)	5.60 m	128.5 (CH)
5	1.45 m	24.9 (CH_2_)	5.91 dt (15.0, 7.0)	134.3 (CH)
6	1.28–1.40, m	28.7–29.2 (CH_2_)	2.08 m	31.8 (CH_2_)
7	1.28–1.40, m	28.7–29.2 (CH_2_)	1.27–1.42, m	28.4–29.7 (CH_2_)
8	1.28–1.40, m	28.7–29.2 (CH_2_)	1.27–1.42, m	28.4–29.7 (CH_2_)
9	1.28–1.40, m	28.7–29.2 (CH_2_)	1.27–1.42, m	28.4–29.7 (CH_2_)
10	1.51 m	28.4 (CH_2_)	1.48 m	28.4–29.7 (CH_2_)
11	2.21 ddd (7.0, 7.0, 2.0)	18.7 (CH_2_)	2.14 m	18.7 (CH_2_)
12		86.7 (C)		81.0 (C)
13		79.6 (C)		77.3 (C)
14	5.28 dd (5.0, 1.5)	58.6 (CH)	3.03 dd (6.5, 5.5)	17.6 (CH_2_)
15	5.71 dd (5.0, 1.5)	132.0 (CH)	5.59 m	128.0 (CH)
16	5.71 dd (5.0, 1.5)	132.0 (CH)	5.59 m	131.0 (CH)
17	5.28 dd (5.0, 1.5)	58.6 (CH)	5.18 d (7.0)	58.2 (CH)
18		79.6 (C)		79.8 (C)
19		86.7 (C)		86.1 (C)
20	2.21 ddd (7.0, 7.0, 2.0)	18.7 (CH_2_)	2.21	18.7 (CH_2_)
21	1.51 m	28.4 (CH_2_)	1.51 m	28.4–29.7 (CH_2_)
22	1.28–1.40, m	28.7–29.2 (CH_2_)	1.27–1.42, m	28.4–29.7 (CH_2_)
23	1.28–1.40, m	28.7–29.2 (CH_2_)	1.27–1.42, m	28.4–29.7 (CH_2_)
24	1.28–1.40, m	28.7–29.2 (CH_2_)	1.27–1.42, m	28.4–29.7 (CH_2_)
25	1.28–1.40, m	28.7–29.2 (CH_2_)	2.08 m	31.8 (CH_2_)
26	1.45 m	24.9 (CH_2_)	5.91 dt (15.0, 7.0)	134.4 (CH)
27	1.72 m	37.6 (CH_2_)	5.60 m	128.5 (CH)
28	4.38 ddd (6.5, 6.5, 2.0)	62.3 (CH)	4.84 d (6.0)	62.8 (CH)
29		85.0 (C)		83.3 (C)
30	2.47 d (2.0)	72.8 (CH)	2.57 d (2.0)	74.0 (CH)

*^a^* 500 MHz in CDCl_3_; *^b^* 125 MHz in CDCl_3_.

Metabolite **2** was obtained as a colorless oil. Its HRESIMS indicated the molecular formula C_30_H_40_O_3_, with eleven degrees of unsaturation. The IR spectrum suggested the presence of a hydroxyl (3414 cm^−1^) and alkyne (3284 and 2209 cm^−1^) moieties. The ^13^C NMR data of **2** (Table 1) showed the presence of 30 carbon signals, which were assigned with the assistance of the DEPT spectrum to eight acetylenic carbons [δ 86.1, 83.3 (2C), 81.0, 79.8, 77.3, and 74.0 (2C)], three oxycarbons [δ 62.8 (2C) and 58.2], six olefinic carbons [δ 134.4, 134.3, 131.0 128.5 (2C) and 128.0] and thirteen methylenes. Comparison of the ^1^H and ^13^C NMR data (Table 1) of compounds **2** and **4** showed that the structure of **2** is similar to that of **4**, with the exception of signals assigned to C-14. The oxymethine signal (δ_H_ 5.26, 1H, s; δ_C_ 58.5) in **4** was replaced by a methylene signal (δ_H_ 3.03, 1H, dd, *J* = 6.5, 5.5 Hz; δ_C_ 17.6) in **2**. Furthermore, the Nuclear Overhauser Effect (NOE) correlation between H_2_-14 and oxymethine proton H-17 indicated a *Z* configuration for the double bond at C-15/C-16 (Figure 3). These results, together with detailed ^1^H–^1^H COSY and HMBC correlations (Figure 3), established the structure of **2** as the 14-deoxy derivative of **4**, and the metabolite was named petrosianyne B.

The cytotoxic activity of compounds **1**–**5** against the proliferation of a limited panel of cancer cell lines, including CCRF-CEM, MOLT-4, K-562, DLD-1, LNCaP and T-47D cells, was evaluated (Table 2). Compounds **1** and **3**–**5** exhibited significant cytotoxic activity against three cancer cells, CCRF-CEM, MOLT-4 and K-562 (IC_50_ < 4 μg/mL), and compound **1** also exhibited significant cytotoxicity against LNCaP and T-47D. In addition, compound **3** exhibited significant cytotoxicity toward DLD-1 and LNCaP cell lines. Compound **2** was not cytotoxic (IC_50_ > 4 μg/mL) toward the above six cancer cell lines. According to the structures of **1**–**4**, compound **2**, the 14-deoxy derivative of **4**, has IC_50_ values of >4 μg/mL against the above carcinoma cell lines; therefore, it was suggested that the hydroxy group of C-14 is important for the cytotoxic activity of compounds **1**–**4**.

**Table 2:** Cytotoxicities (IC_50_ μg/mL) of compounds **1**–**5**.

Compound	Cell Lines					
	CCRF-CEM	MOLT-4	K-562	DLD-1	LNCaP	T-47D
**1**	0.6 ± 0.2	<0.1	3.3 ± 1.2	NA ^b^	3.2 ± 1.2	0.3 ± 0.1
**2**	6.3 ± 2.5	5.7 ± 1.2	7.8 ± 3.2	NA	NA	NA
**3**	0.8 ± 0.2	0.7 ± 0.1	3.4 ± 1.1	<0.1	3.2 ± 1.4	NA
**4**	2.5 ± 1.8	3.0 ± 1.6	4.0 ± 2.3	7.4 ± 3.1	NA	4.4 ± 1.1
**5**	0.4 ± 0.1	0.7 ± 0.5	3.4 ± 0.4	3.7 ± 1.8	5.9 ± 2.2	7.3 ± 2.2
**Doxorubicin** ^a^	<0.1	<0.1	<0.1	5.8 ± 2.1	1.9 ± 1.1	<0.1

*^a^* Clinical anticancer drug used as a positive control; *^b^* NA, not active at 10 μg/mL.

## 3. Experimental Section

### 3.1. General Procedures

Optical rotation values were measured with a Jasco P-1010 digital polarimeter (Jasco, Tokyo, Japan). IR spectra were recorded on a Varian Digilab FTS 1000 Fourier transform infrared spectrophotometer (Varian Inc., Palo Alto, CA, USA). The NMR spectra were recorded on a Varian Mercury Plus 400 FT-NMR or Varian Unity INOVA 500 FT-NMR, (Varian Inc., Palo Alto, CA, USA) instrument at 400 MHz (or 500 MHz) for ^1^H NMR and 100 MHz (or 125 MHz) for ^13^C NMR in CDCl_3_. ESIMS were obtained with a Bruker APEX II mass spectrometer (Bruker Daltonics, Billerica, MA, USA). Gravity column chromatography was performed on silica gel (230–400 mesh, Merck, Darmstadt, Germany). Thin layer chromatography (TLC) was carried out on precoated Kieselgel 60 F254 (0.2 mm, Merck, Darmstadt, Germany) and spots were visualized by spraying with 10% H_2_SO_4_ solution followed by heating. High-performance liquid chromatography (HPLC) was performed using a system comprised of a Hitachi L-7100 pump (Hitachi, Ltd., Tokyo, Japan) and a Rheodyne 7725 injection port (Rheodyne LLC, Rohnert Park, CA, USA). A preparative normal phase column (Hibar 250 × 21.2 mm, Supelco, silica gel 60, 5 μm, Supelco Inc., Bellefonte, PA, USA) was used for HPLC.

### 3.2. Animal Material

**Figure 4:**
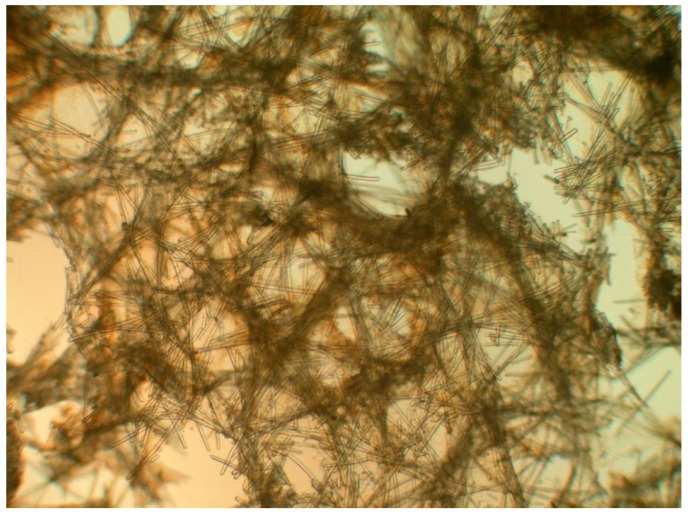
*Petrosia* sp. specimen: Detail of the ectosome, showing foreign materials.

**Figure 5:**
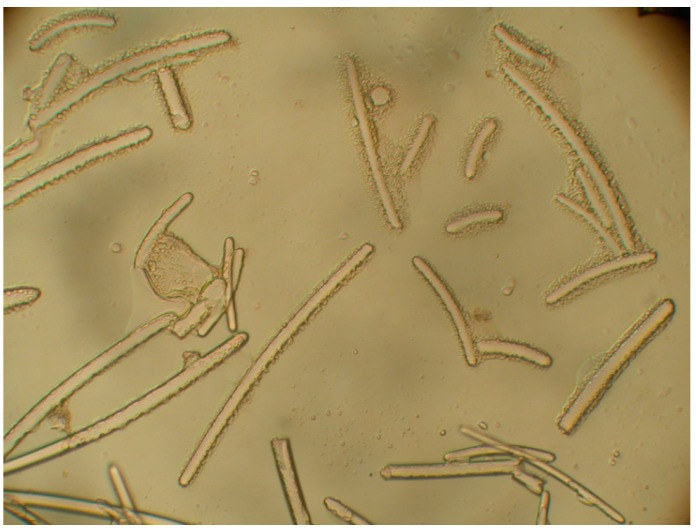
Spicules of the ectosomal periphery observed using an optical microscope.

The sponge *Petrosia* (Vosmaer, 1887) sp. belongs to the class Demospongiae, order Haplosclerida (Topsent 1928), family Petrosiidae (Van Soest, 1980) [18]. It was collected by scuba divers at a depth of 15–20 m from coral reefs off the coast of Pingtung, Taiwan, in February 2013. This sponge is irregular in shape and dark purple in color (Figure 1). The ectosomal skeleton is dense, granular, with a tangential network of single strongyles forming a subrectangular mesh, with free strongyles grouped at the nodes of the network (Figure 4). Abundant abruptly curved microxeas were observed. Choanosomal spicule tracts were slightly differentiated with stout longitudinal tracts and connecting short, irregular tracts of strongyles and free strongyles. Megascleres measured from 188–322 µm and microscleres from 72–100 µm (Figure 5). The sponge was identified as *Petrosia* sp. and a voucher specimen was deposited at the National Museum of Marine Biology and Aquarium, Taiwan (specimen no. SP2013-1). Taxonomic identification was performed by Prof. Li-Lian Liu, National Sun Yat-sen University, Kaohsiung, Taiwan.

### 3.3. Extraction and Separation

The sponge *Petrosia* sp. (1.16 kg fresh weight) was stored frozen and then freeze dried. The freeze-dried material (370 g) was minced and extracted exhaustively with EtOAc (5 × 2 L). The EtOAc extract was evaporated to yield a residue (4.81 g), which was subjected to open column chromatography on silica gel, eluting with *n*-hexane (H)-EtOAc (E) gradient and EtOAc (E)-acetone (A) gradient, to yield 13 fractions. Fraction 9 (450 mg), eluted with *n*-hexane:EtOAc (1:2), was further purified by normal phase HPLC using *n*-hexane:EtOAc (3:1) to afford **5** (12.7 mg). Fraction 10 (720 mg), eluted with pure EtOAc, was separated by normal phase HPLC, using *n*-hexane:EtOAc (3:2) to afford seven subfractions (10A–10G). Subfraction 10B was separated by normal phase HPLC using *n*-hexane:EtOAc (5:2) to afford **2** (5.5 mg). Compounds **1** (2.1 mg), **4** (361.7 mg) and **3** (8.2 mg) were obtained from subfractions 10C−10E by normal phase HPLC using *n*-hexane:EtOAc (2:1), *n*-hexane:EtOAc (3:2) and *n*-hexane:EtOAc (1:1), respectively.

Petrosianyne A (**1**): colorless oil; 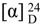 = +82 (*c* 0.2, CHCl_3_); IR (neat) ν_max_ 3425, 3280, 2923, 2225, 1651, 1458 and 1270 cm^−1^; ^1^H and ^13^C NMR data, see Table 1; ESIMS *m*/*z* 491 [100, (M + Na)+]; HRESIMS *m*/*z* 491.3135 (calcd. for C_30_H_44_O_4_Na, 491.3137).

Petrosianyne B (**2**): colorless oil; 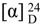 = +37 (*c* 0.2, CHCl_3_); IR (neat) ν_max_ 3414, 3284, 2926, 2854, 2209, 2096, 1647 and 1237 cm^−1^; ^1^H and ^13^C NMR data, see Table 1; ESIMS *m*/*z* 471 [100, (M + Na)+]; HRESIMS *m*/*z* 471.2873 (calcd. for C_30_H_44_O_3_Na, 471.2875).

15,16-Dihydropetrosianyne (**3**): colorless oil; 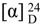 +18 (*c* 2.0, CHCl_3_); [lit. 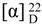 +10 (*c* 0.14, CHCl_3_)] [16].

Petrosynol (**4**): colorless oil; 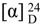 +102 (*c* 2.0, CHCl_3_); [lit. 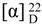
+111 (*c* 1.29, CHCl_3_)] [17].

### 3.4. Supplementary Files

HR-ESI-MS, ^1^H NMR, and ^13^C NMR spectra for the two new compounds (**1** and **2**) as well as the ^1^H NMR, and ^13^C NMR spectra for compounds **3**–**5** are available as Supplementary Information.

### 3.5. Cytotoxicity Testing

The MTT assay was performed as described previously with some modification [19]. Cytotoxicity assays of compounds **1**–**5** were conducted against human T cell lymphoblast-like cell line (CCRF-CEM), human T lymphoblast, acute lymphoblastic leukemia (MOLT-4), human chronic myelogenous leukemia (K-562), human colon adenocarcinoma (DLD-1), human prostate carcinoma (LNCaP) and human hormone-dependent breast cancer (T-47D) using a MTT colorimetric method. The tested human cancer cell lines were seeded at 4 × 10^4^ per well in 96-well culture plates before treatment with different concentrations of the tested compounds. Compounds **1**–**5** were dissolved in DMSO (less than 0.02%) and made immediately to 1.25, 2.5, 5, 10 and 20 μg/μL prior to the experiments. After treatment for 72 h, the cytotoxicities of the tested compounds were determined using a MTT cell proliferation assay (thiazolyl blue tetrazolium bromide, Sigma-M2128, Sigma-Aldrich, St. Louis, MO, USA). The MTT is reduced by the mitochondrial dehydrogenases of viable cells to a purple formazan product. The MTT-formazan product was dissolved in DMSO. Light absorbance values (OD = OD_570_ − OD_620_) were recorded at wavelengths of 570 and 620 nm using an ELISA reader (Anthos labtec Instrument, Salzburg, Austria) to calculate the concentration that caused 50% inhibition (IC_50_), *i.e.*, the cell concentration at which the light absorbance value of the experimental group was half that of the control group. These results were expressed as a percentage of the control ± SD established from *n* = 4 wells per one experiment from three separate experiments. 

## 4. Conclusions

In recent years, a series of new polyacetylenes was isolated from a marine sponge of the *Petrosia* sp [5,7,8,9,10,11,12,13,14,15,16,20,21,22,23,24,25].Some of these polyacetylenic compounds have been found to possess significant cytotoxicity [5,7,9,14,15,20,21,24].Our continued investigation of the chemical constituents of the sponge *Petrosia* sp. has led to the isolation of two new polyacetylenic compounds (**1** and **2**), along with three known polyacetylenes (**3**–**5**). Compounds **1** and **3**–**5** exhibited significant cytotoxicity against three cancer cells, CCRF-CEM, MOLT-4 and K-562 (IC_50_ < 4 μg/mL), and compound **1** also exhibited significant cytotoxicity against LNCaP and T-47D. In addition, compound **3** exhibited significant cytotoxicity toward DLD-1 and LNCaP cell lines. These results suggested that polyacetylenes as a unique class of secondary metabolites represent a treasure trove for potential cytotoxic compounds.

## Supplementary Materials

Supplementary Tables can be found at: http://www.mdpi.com/1422-0067/15/9/16511/s1.

## Supplementary Files

Supplementary File 1
