# Supplementary material for: Structure Elucidation and Cytotoxic Evaluation of New Polyacetylenes from a Marine Sponge Petrosia sp
Source: Int J Mol Sci. 2014 Sep 18;15(9):16511–21. doi: 10.3390/ijms150916511 (PMC4200862; doi:10.3390/ijms150916511)

Supplementary Information

Figure S1. HR-ESI-MS spectrum of **1**.

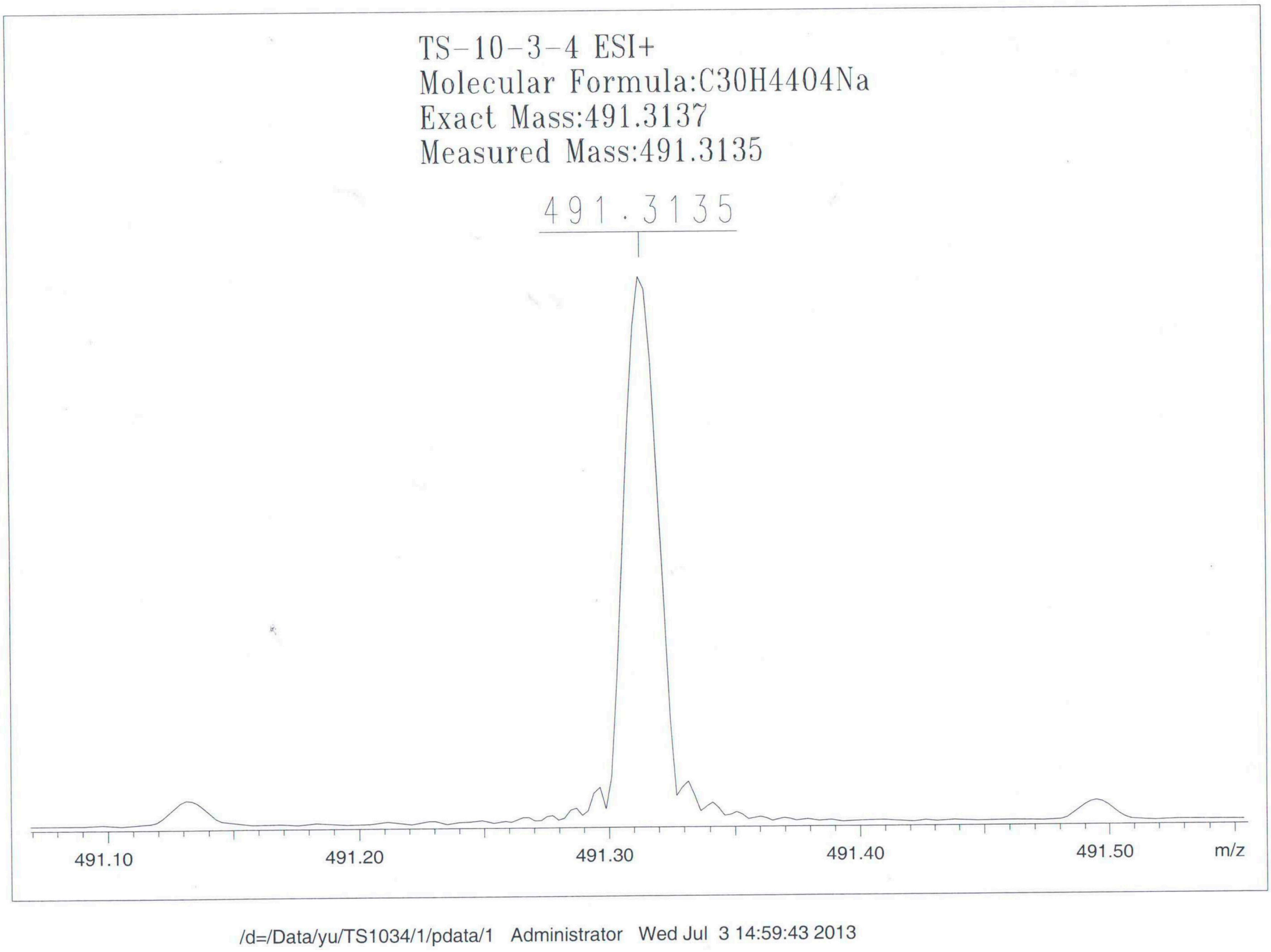

Figure S2. ^{1}H NMR spectrum of 1 in $CDCl_3$ at 500 MHz.

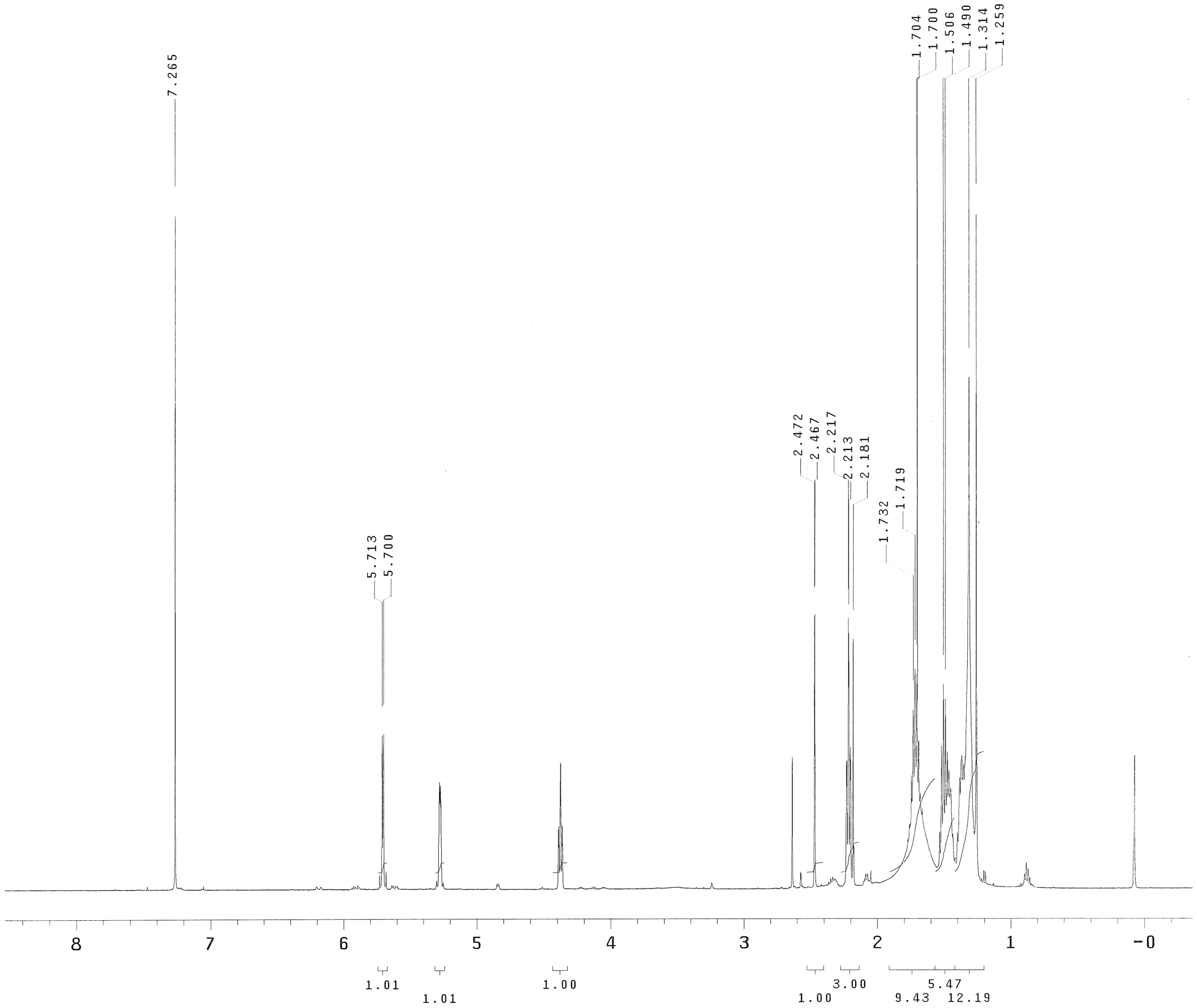

Figure S3. ^{13}C NMR spectrum of **1** in $CDCl_3$ at 125 MHz.

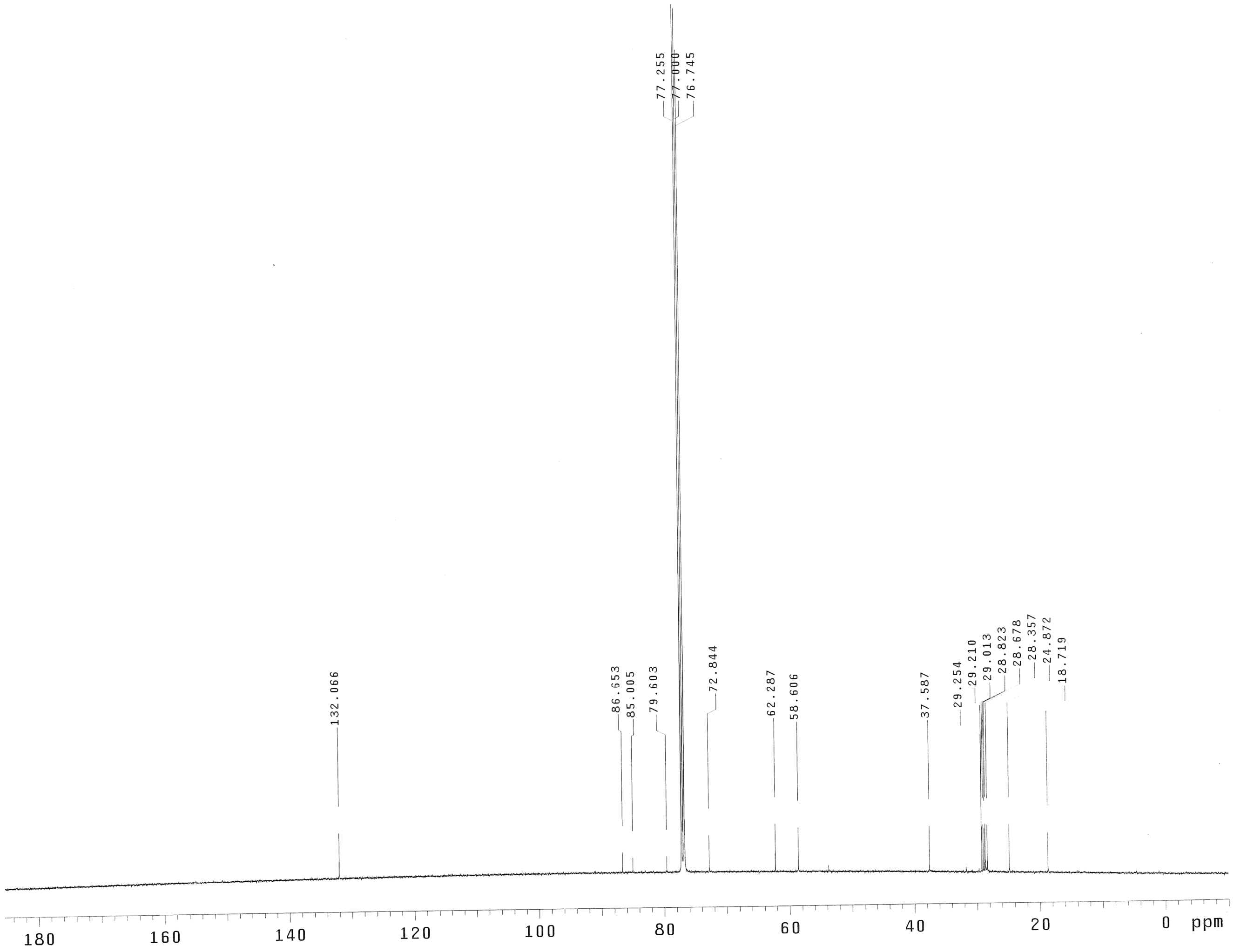

Figure S4. HR-ESI-MS spectrum of **2**.

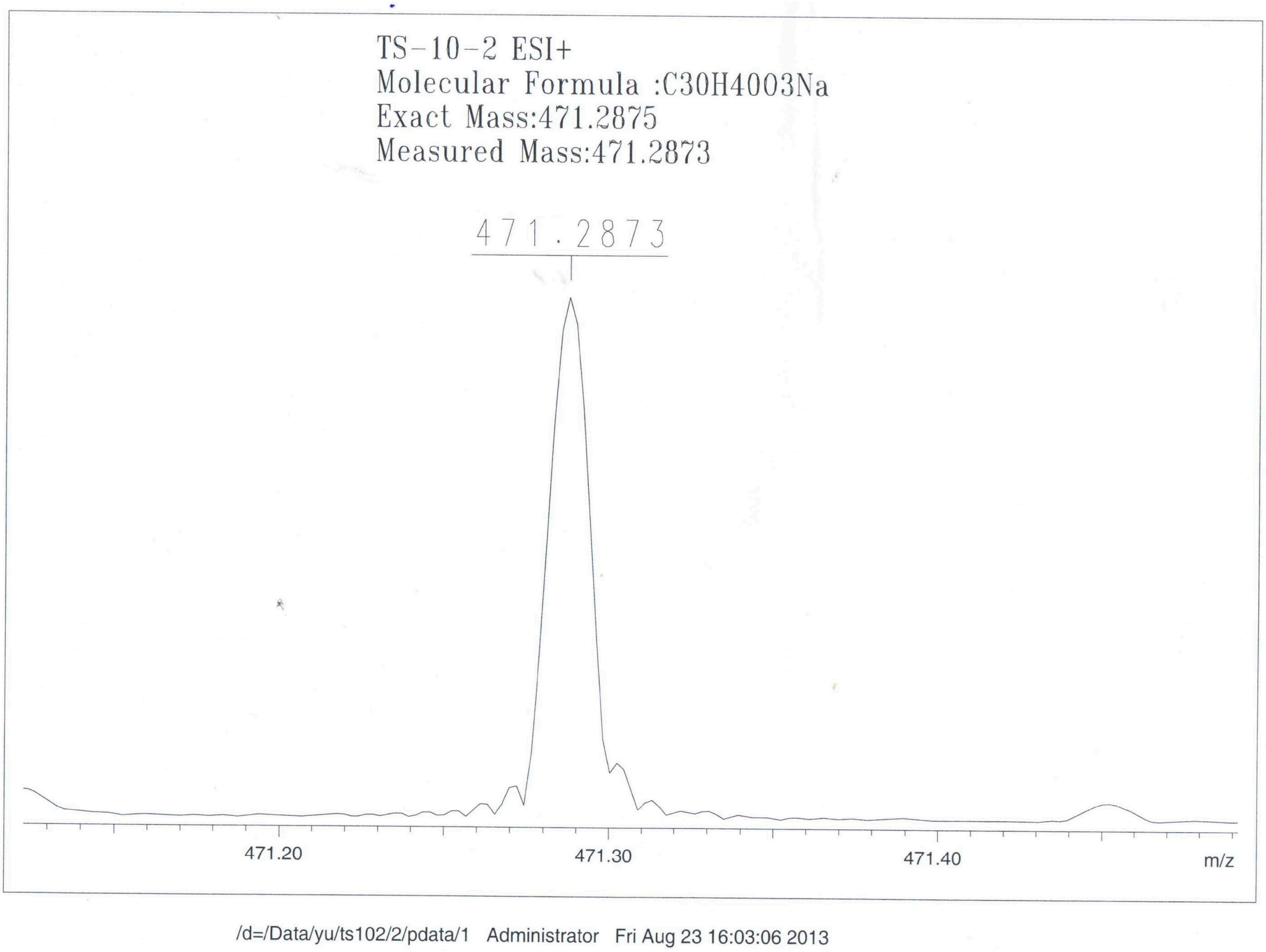

Figure S5. ^{1}H NMR spectrum of **2** in $CDCl_3$ at 500 MHz.

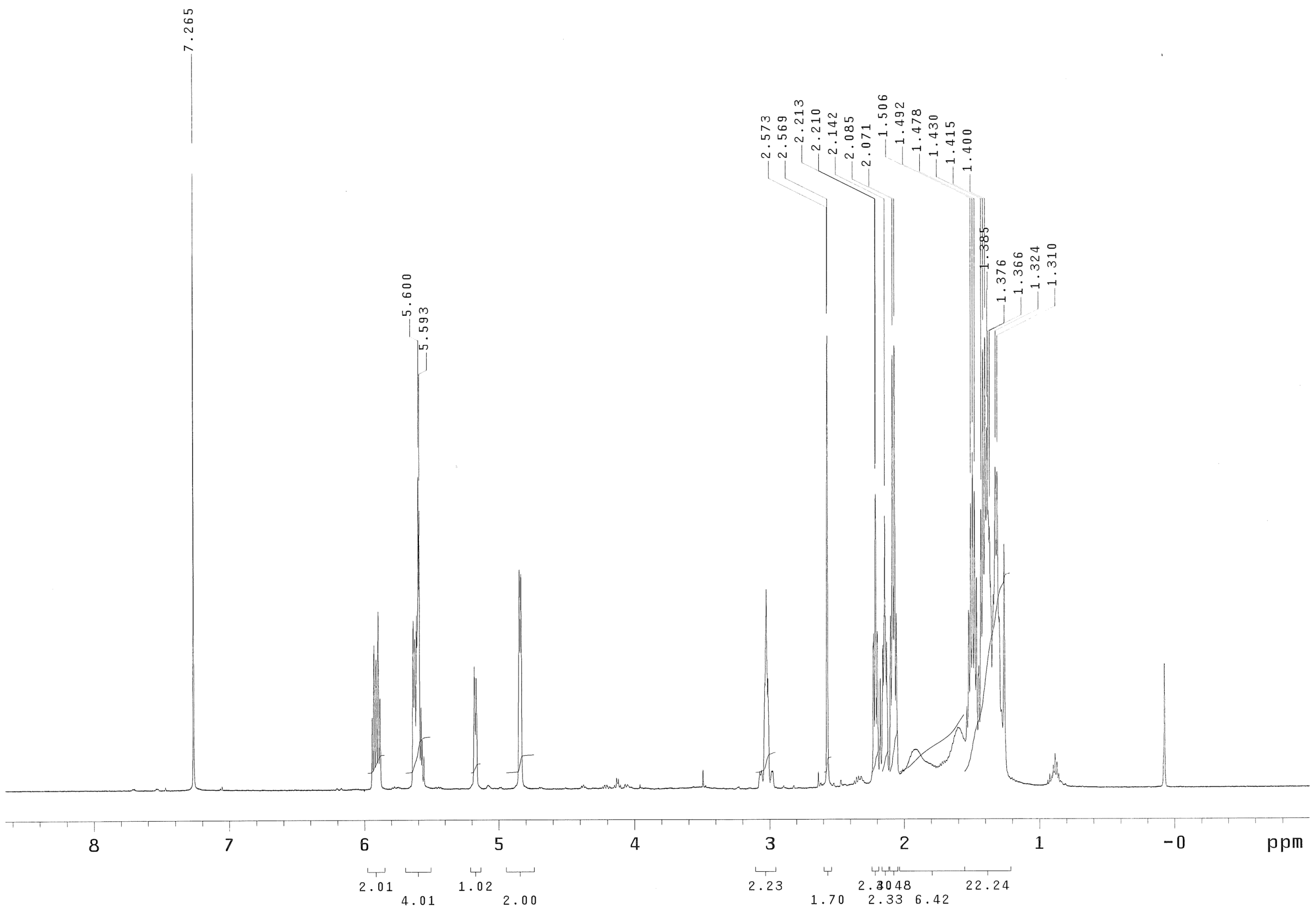

Figure S6. ^{13}C NMR spectrum of **2** in $CDCl_3$ at 125 MHz.

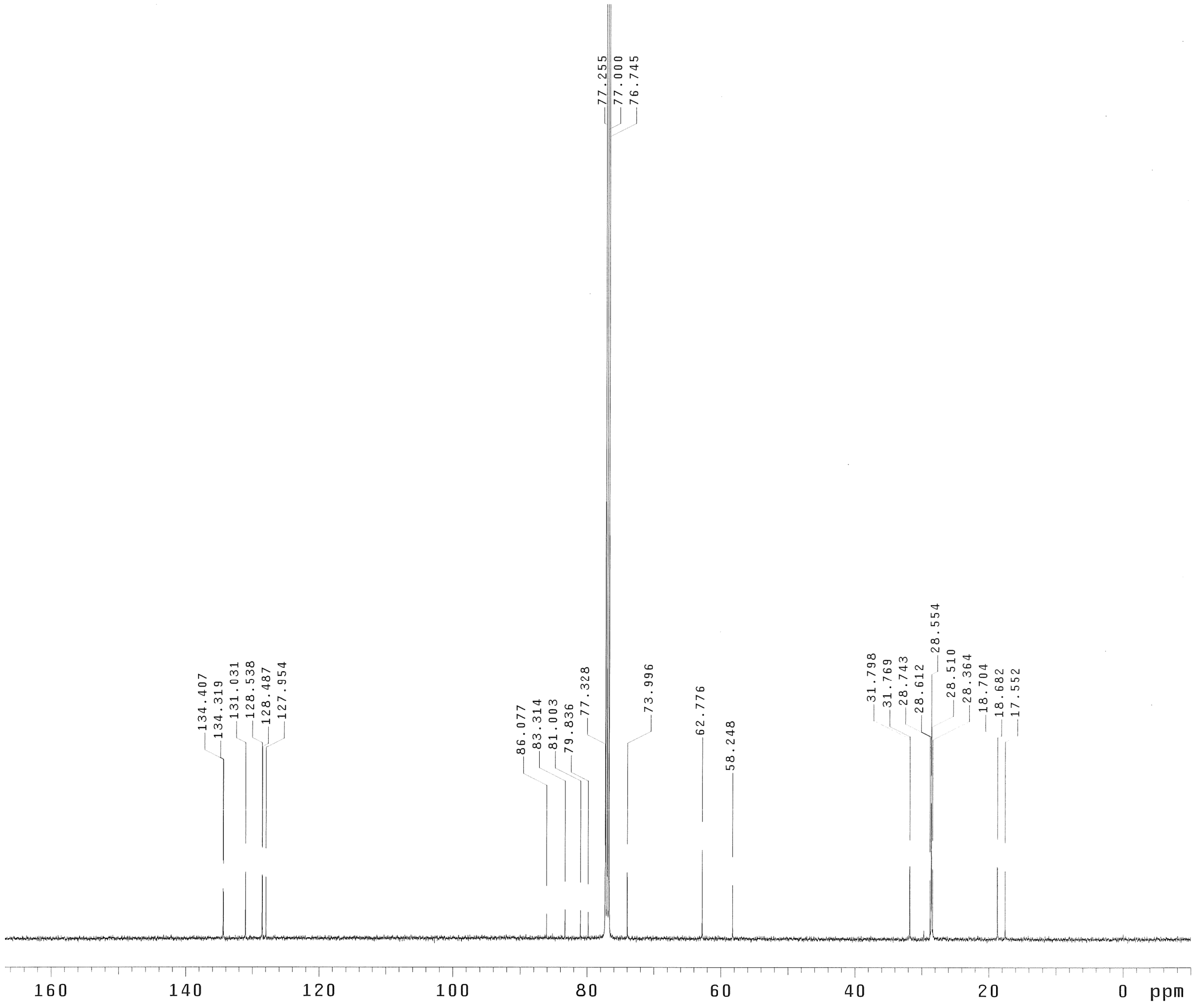

Figure S7. ^{1}H NMR spectrum of **3** in $CDCl_3$ at 500 MHz.

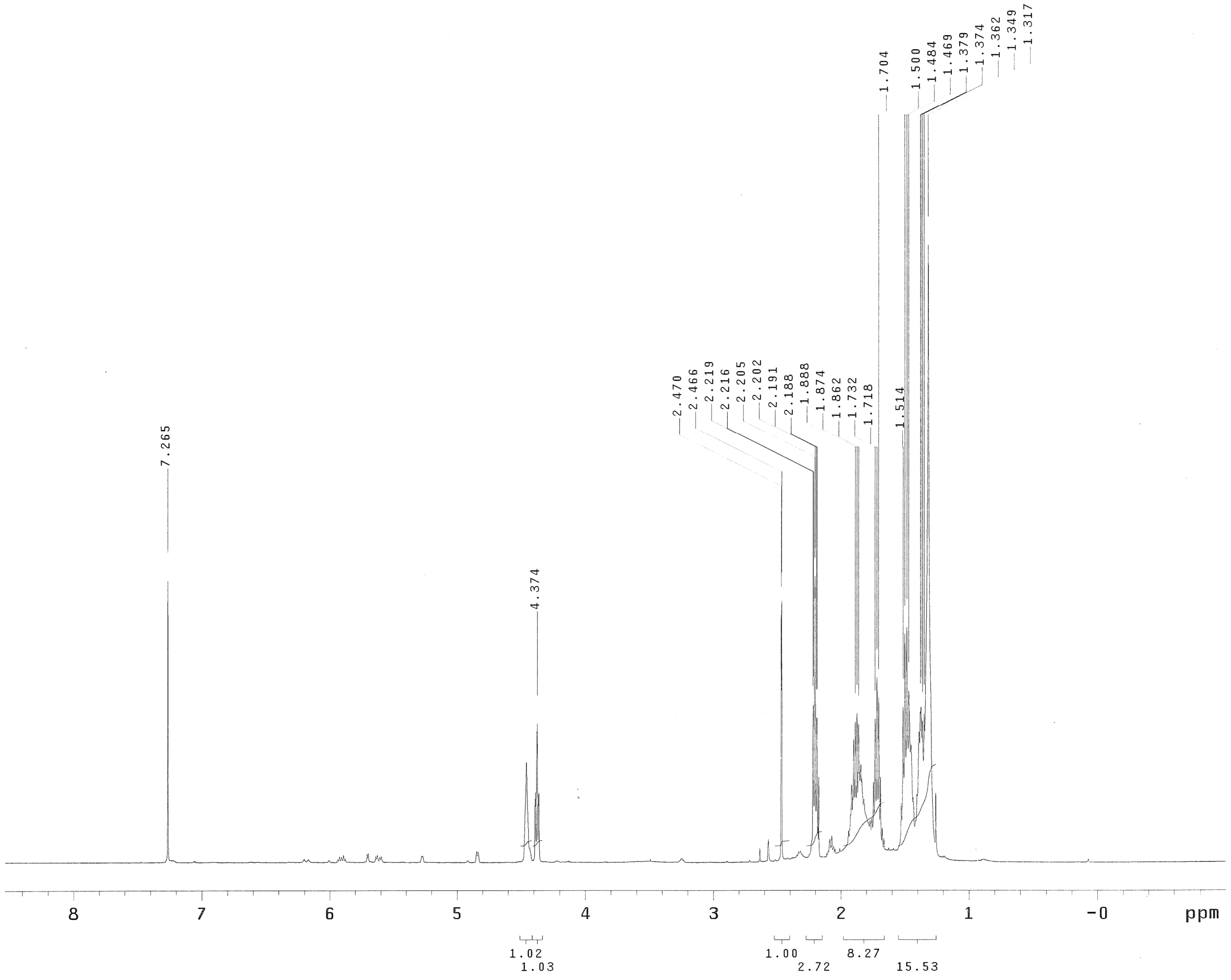

Figure S8. ^{13}C NMR spectrum of **3** in $CDCl_3$ at 125 MHz.

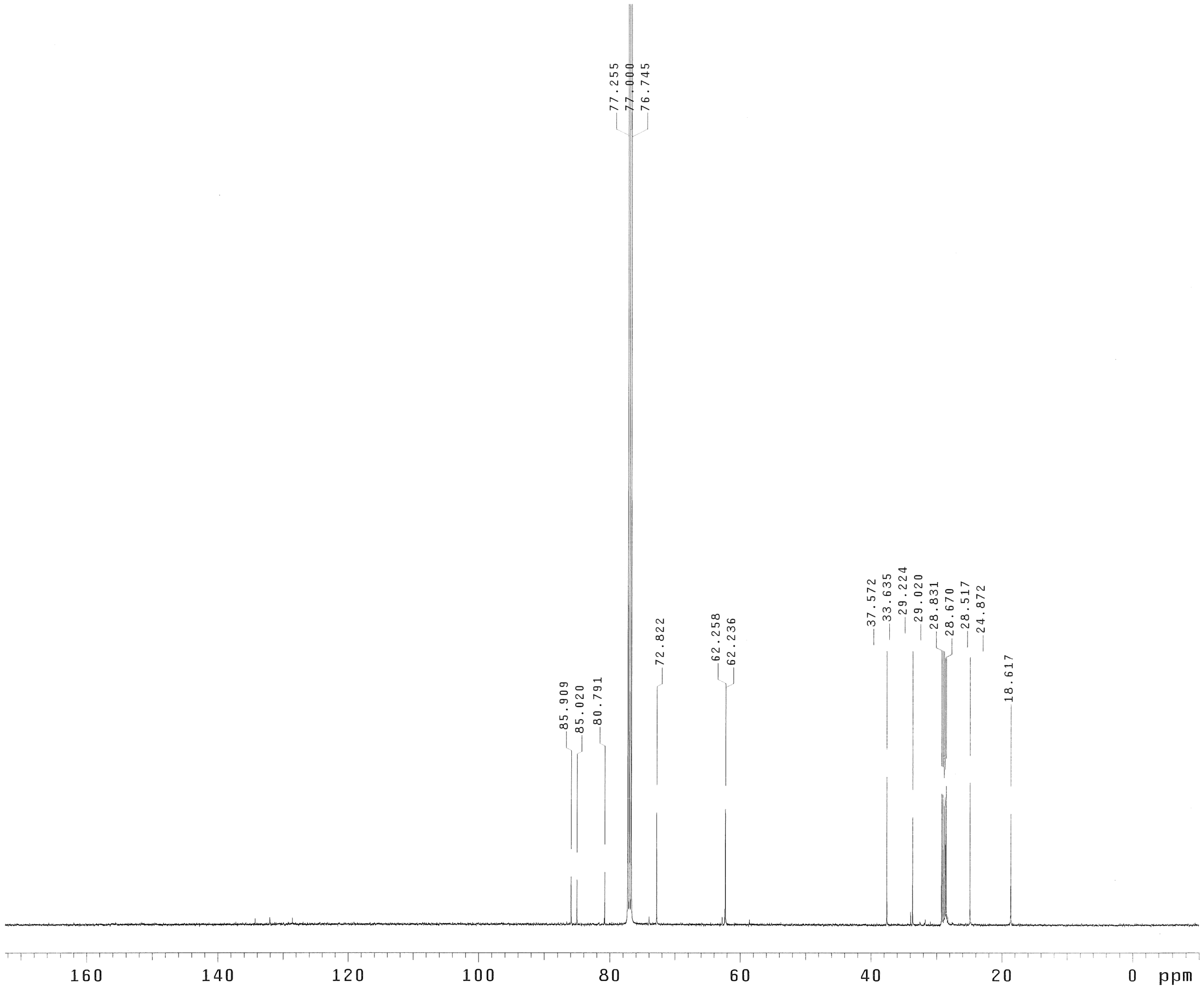

Figure S9. ^{1}H NMR spectrum of **4** in $CDCl_3$ at 400 MHz.

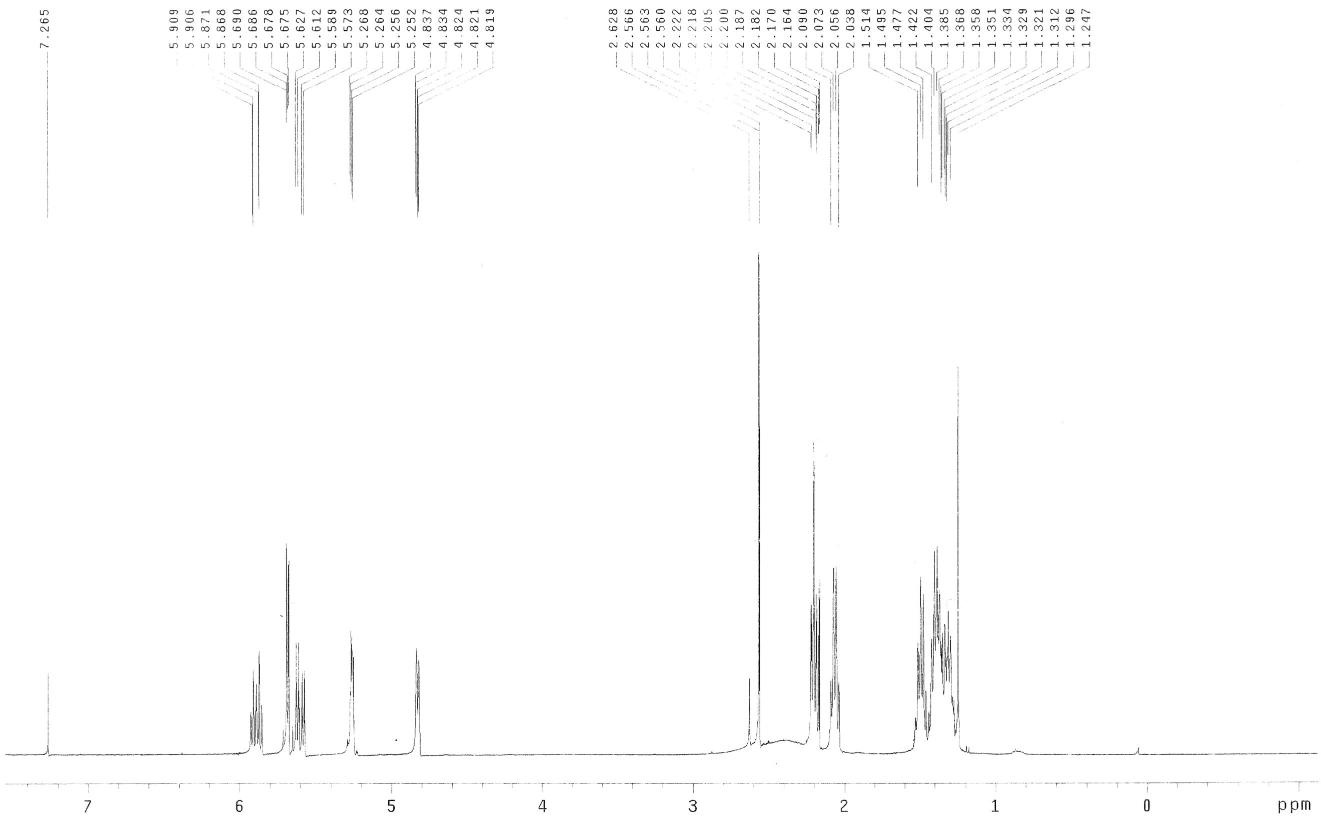

Figure S10. ^{13}C NMR spectrum of **4** in $CDCl_3$ at 100 MHz.

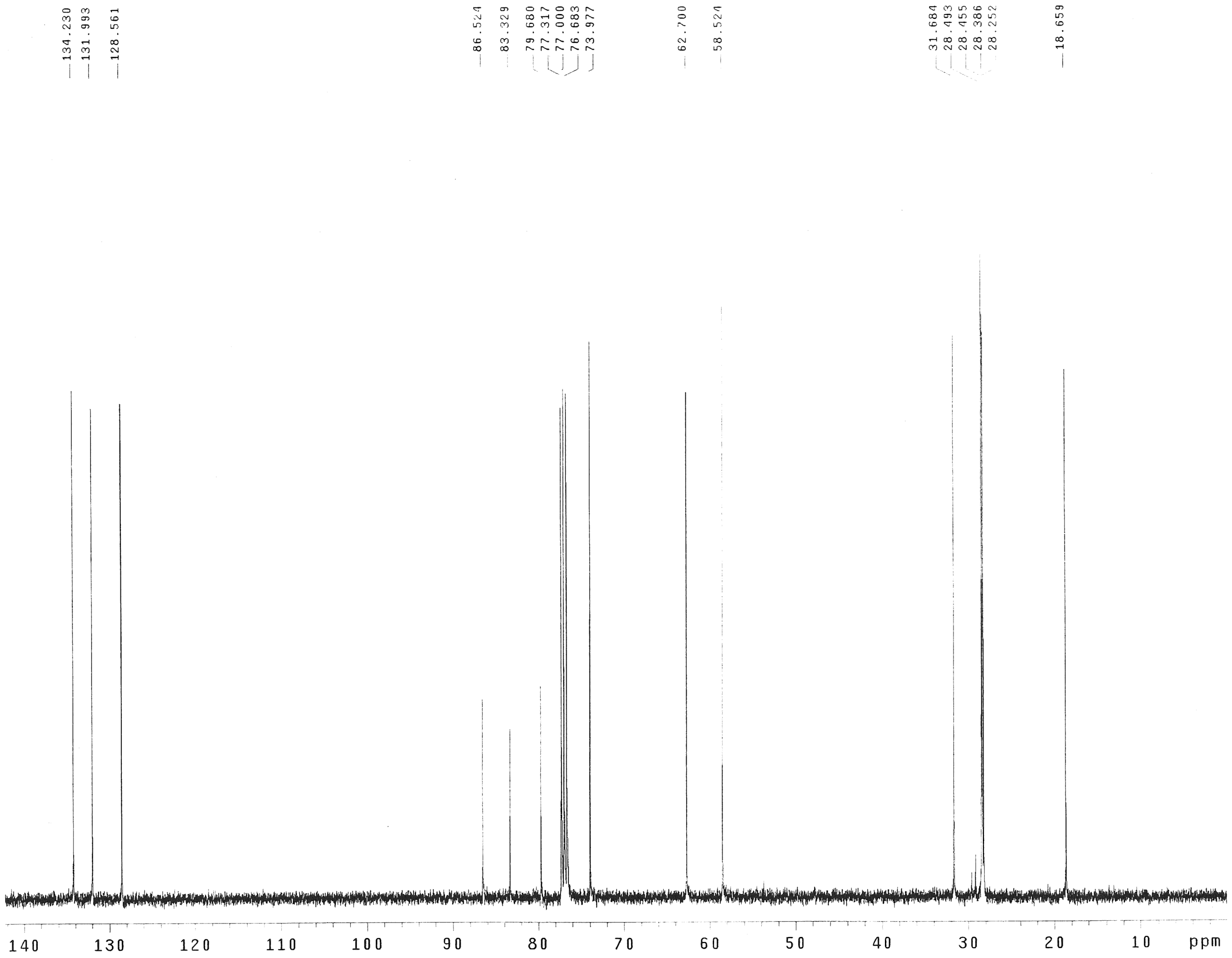

Figure S11. ^{1}H NMR spectrum of **5** in $CDCl_3$ at 500 MHz.

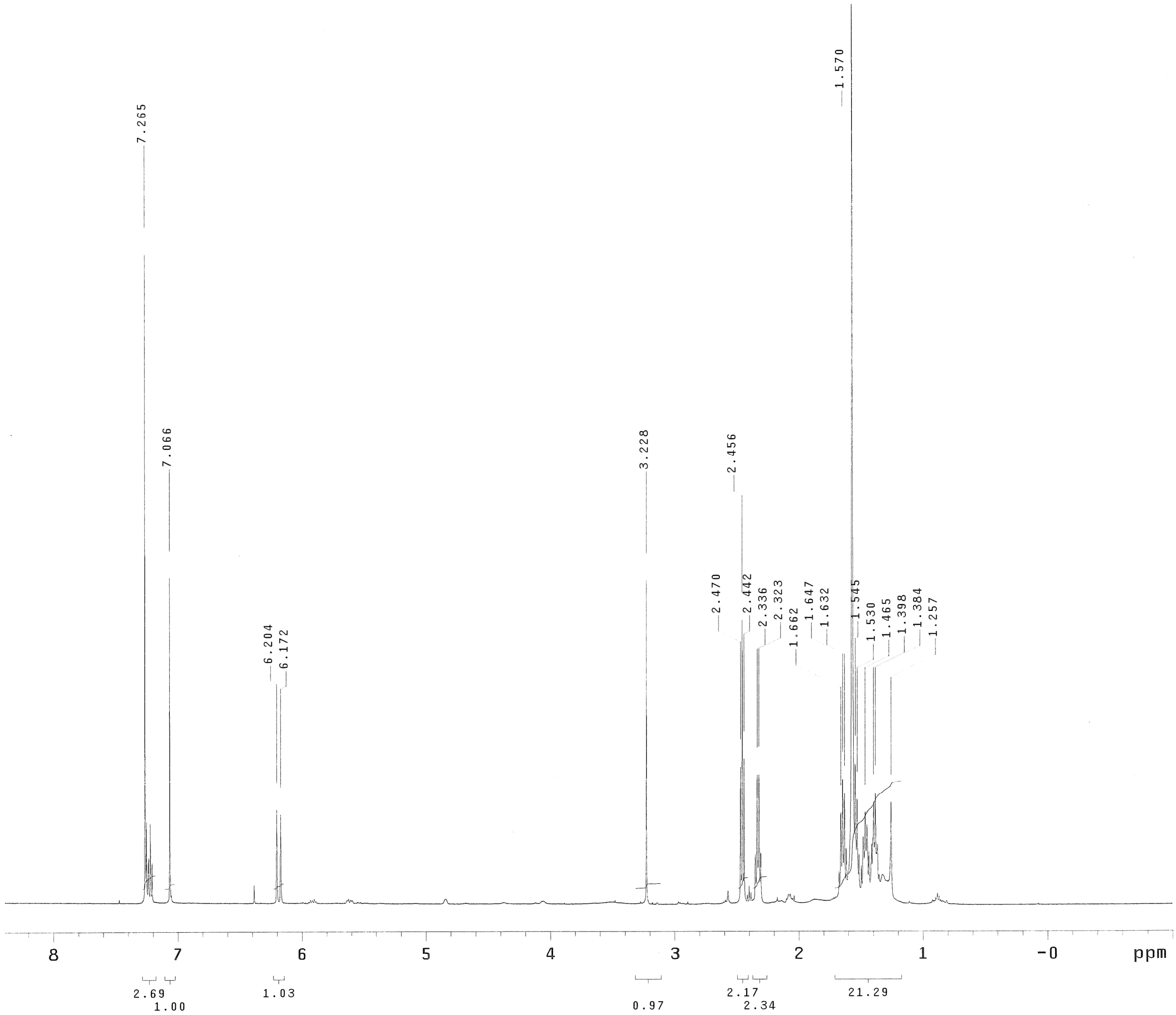

Figure S12. ^{13}C NMR spectrum of **5** in $CDCl_3$ at 125 MHz.

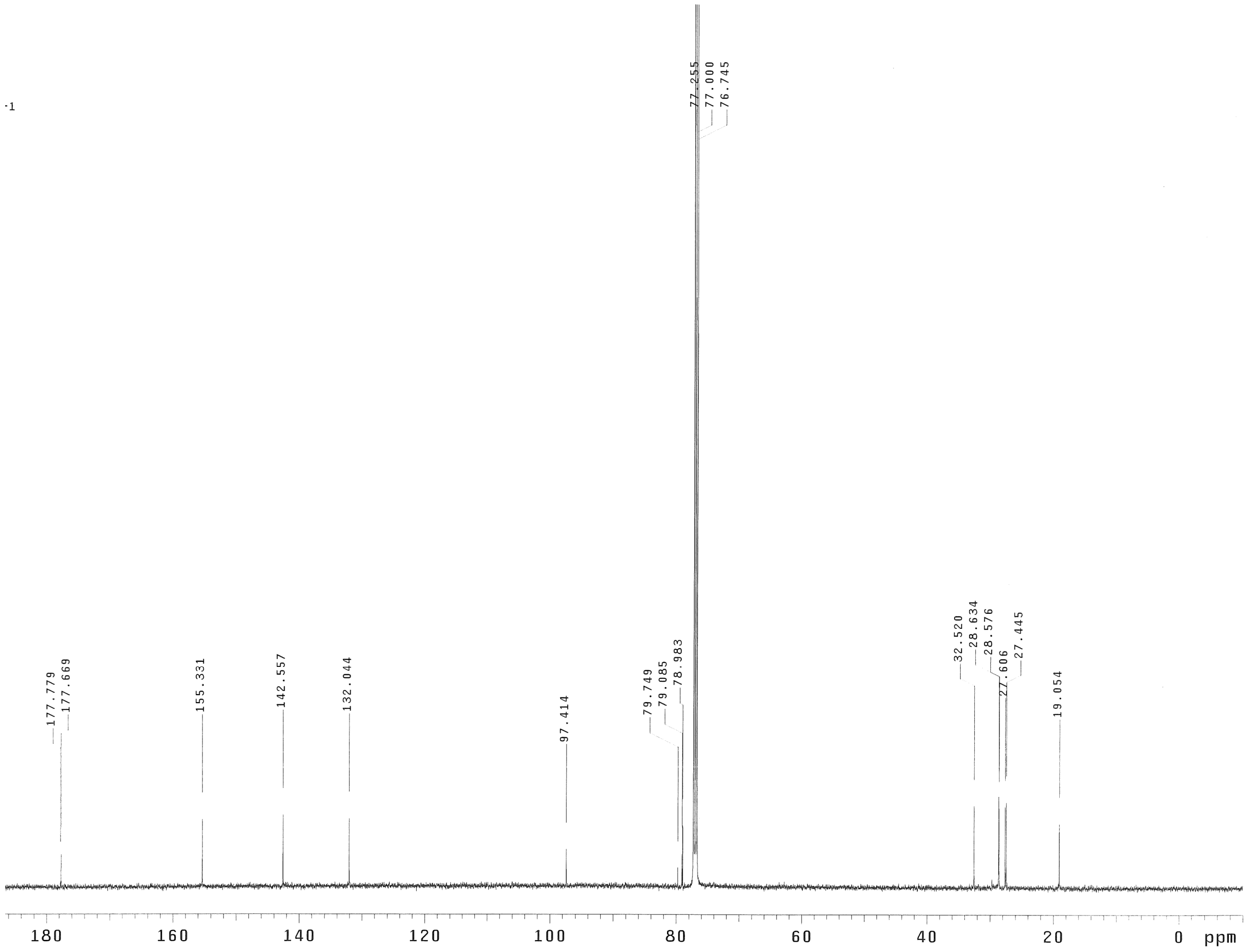

Supplement: Supplementary File 1 [file ijms-15-16511-s001.pdf]
